# Effect of Social Rank upon Estrus Induction and Some Reproductive Outcomes in Anestrus Goats Treated With Progesterone + eCG

**DOI:** 10.3390/ani10071125

**Published:** 2020-07-02

**Authors:** Santiago Zuñiga-Garcia, Cesar A. Meza-Herrera, Adela Mendoza-Cortina, Julio Otal-Salaverri, Carlos Perez-Marin, Noé M. Lopez-Flores, Evaristo Carrillo, Guadalupe Calderon-Leyva, Ulises N. Gutierrez-Guzman, Francisco G. Veliz-Deras

**Affiliations:** 1 Unidad Laguna, Universidad Autónoma Agraria Antonio Narro, Periférico Raúl López Sánchez y Carretera a Santa Fe, 27054 Torreón, Coahuila, Mexico; s_zuniga83@hotmail.com (S.Z.-G.); gcalderon06@hotmail.com (G.C.-L.); 2 Unidad Regional Universitaria de Zonas Áridas, Universidad Autónoma Chapingo, 35230 Bermejillo, Durango, Mexico; cmeza2020@hotmail.com (C.A.M.-H.); adela.mendoza@chapingo.uruza.edu.mx (A.M.-C.); noe.lopez.flores@hotmail.com (N.M.L.-F.); 3 Departamento de Producción Animal, Facultad de Veterinaria, Universidad de Murcia, 30100 Murcia, Spain; juotal@um.es; 4 Instituto de Estudios de Posgrado, Facultad de Veterinaria, Universidad de Córdoba, 14014 Córdoba, Spain; pv2pemac@uco.es; 5 Instituto Tecnológico de Torreón, 27170 Torreón, Coahuila, Mexico; evaristocarrillo@yahoo.com.mx; 6 Facultad de Agricultura y Zootecnia, Universidad Juárez del Estado de Durango, 35111 Venecia, Durango, Mexico; ulisesnoelg@yahoo.com.mx

**Keywords:** goats, social ranks, anestrus season, estrus induction protocol, reproductive efficiency

## Abstract

**Simple Summary:**

The potential effect of social rank [R] (high—HSR; medium—MSR; low—LSR) in anestrus goats subjected to an estrus induction protocol (EIP) primed with progesterone (P4) and receiving a differential equine chorionic gonadotropin (eCG) dose [D] (D100 vs D350) upon some reproductive outcomes in crossbred dairy goats under intensive stall-fed conditions was evaluated. Response variables included estrus induction (EI, %), latency to estrus (LAT, h), duration of estrus (DUR, h), ovulation (OVU, %), ovulation rate (OR, n), corpus luteum size (CLS, cm), pregnancy (PREG, %), kidding (KIDD, %), and litter size (LS, n). Most of the response variables were positively affected by social rank, favoring to the HSR goats (i.e., EI %, DUR h, OVU %, OR n, and CLS cm). In addition, increased OR and PREG occurred in the HSR + D350 group, while D350 increased LS, irrespective of R. Interestingly, since no differences regarding LAT, DUR, OVU, CLS, PREG, and KIDD occurred between D350 and D100, the obtained values support the use of a reduced level of exogenous hormones to induce and generate out-of-season reproductive efficiency.

**Abstract:**

We evaluated the possible role of the social rank [R] (i.e., low—LSR, middle—MSR, or high—HSR) in anestrus goats exposed to a P4 + eCG [D] (i.e., 100 or 350 IU) estrus induction protocol (EIP). Adult, multiparous (two to three lactations), multiracial, dairy-type goats (Alpine–Saanen–Nubian x Criollo goats (*n* = 70; 25°51′ North) managed under stall-fed conditions were all ultrasound evaluated to confirm anestrus status while the R was determined 30 d prior to the EIP. The variables of estrus induction (EI, %), estrus latency (LAT, h), estrus duration (DUR, h), ovulation (OVU, %), ovulation rate (OR, n), corpus luteum size (CLS, cm), pregnancy (PREG, %), kidding (KIDD, %), and litter size (LS, n) as affected by R, D, and the R × D interaction, were evaluated. While OVU and CLS favored (*p* < 0.05) HSR (96% and + 1.04 ± 0.07 cm), an increased (*p* < 0.05) LS occurred in D350 vs. D100 (2.06 ± 0.2 vs. 1.36 ± 0.2); neither R nor D affected (*p* > 0.05; 38.5%) KIDD. However, EI, LAT, DUR, OR, and PREG were affected by the R × D interaction. The HSR group had the largest (*p* < 0.05) EI % and DUR h, irrespective of D. The shortest (*p* < 0.05) LAT occurred in D350, irrespective of R. While the largest (*p* < 0.05) OR occurred in HSR and MSR within D350, the HSR + D350 group had the largest PREG (*p* < 0.05). These research outcomes are central to defining out-of-season reproductive strategies designed to attenuate seasonal reproduction in goats.

## 1. Introduction

The goat is a gregarious species [1,2,3] with a well-defined hierarchical structure and different social ranks coexist [2,4]. These social behavioral differences give individuals different opportunities not only to survive [2,3,5], but to express reproductive fitness and success in small ruminants, in either males or females [2,4,6,7]. Indeed, in mountain goats (*Oreamnos americanus*), the dominant females displayed a greater reproductive success regarding the number of kids born as compared to the subordinate goats [8]. Furthermore, in females of the Spanish ibex (*Capra pyrenaica*) in captivity, the dominant goats exhibited a greater number of estrus cycles and higher pregnancy rates, as compared to the subordinate goats [9]. Certainly, when exposed to the male effect, the hierarchically dominant Kashmir female goats responded in the first 4 h with not only higher luteinizing hormone (LH) pulses but also greater success when expressing estrus [7]. Furthermore, high socially ranked female goats exposed to the male effect had a significantly earlier ovulation and greater pregnancy rates compared to low socially ranked goats. Indeed, hierarchically dominant goats had longer contact periods with males, which were thus able to promote an enhanced sexual bio-stimulation and, therefore, induced increased reproductive outcomes [1]. Certainly, a longer male-to-female interaction during the male effect process promotes an increased secretion of luteinizing hormone (LH), mainly in dominant goats [7,10]. Moreover, when exposed to an estrus synchronization protocol, high-ranking goats had higher progesterone secretion during the maternal recognition stage of the pregnancy process relative to low or medium social ranks [11]. Similarly, in a superovulation protocol, the dominant goats presented a greater number of corpus lutea [12].

On the other hand, the goat is considered a seasonal short-day polyestrus breeder, which allows it to mate and give birth in a defined season of the year [13,14]. This reproductive seasonality causes milk, cheese, and meat to concentrate in some specific months of the year, causing economic losses to both producers [15] and industrializers [16,17]. To resolve this problem and to induce out-of-season sexual activity, diverse estrus-inducing hormone protocols have been used, obtaining interesting results regarding both estrus induction and ovulation [18]. Among the hormonal protocols to control reproductive activity in goats, are those progestogen-based protocols involving natural progesterone [19], fluorogestone acetate [20], or medroxyprogesterone acetate [21]. Those estrus induction protocols using progestogens are normally accompanied by the use of gonadotropins, such as human chorionic gonadotropin (hCG) [22] or equine chorionic gonadotropin (eCG) [18].

Currently, animal production is aimed at sustainability, with a strict regulation regarding the use of exogenous hormones [18]. Therefore, any attempt to decrease the use of exogenous hormones for reproductive control is not only an interesting option but also a claimed area of research. The use of 100 IU of hCG has been shown to effectively induce reproductive activity in anestrus goats, obtaining similar results with conventional higher doses [12,23]. Nonetheless, in goats, there is little evidence regarding the relationship between social rank and the induction of sexual activity in goats subjected to a hormonal estrus-inducing protocol. Building on such findings, we hypothesized a differential response according to social rank in anestrus goats subjected to an intramuscular progesterone + eCG estrus induction protocol; while a better reproductive response is expected to occur in the HSR goats, we also propose that a similar reproductive performance will be observed in goats receiving either a high or a low eCG dose (i.e., 100 or 350 IU); this study aimed to answer such an inquiry.

## 2. Material and Methods

### 2.1. General

All the experimental procedures, methods, and the management of the trial experimental units used in this study were compliant with the guidelines for the ethical use, care, and welfare of animals in research at international [24] and national [25] levels, with institutional approval reference number UAAAN-UL-18-3059.

### 2.2. Location, Environmental Conditions, Animals, and their Management

**Figure 1:**
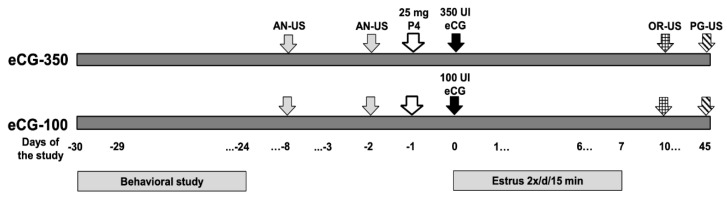
Schematic representation of the experimental protocol. In February, a behavioral study was carried out to define the social ranks: high (HSR), middle (MSR), or low (LSR) social rank. Then, all goats were exposed to an estrus induction protocol, in order to induce reproductive activity during the natural anestrus season in Northern Mexico (March; 25°51’ North). All goats were primed with progesterone (P4) and received different doses of equine chorionic gonadotropin (eCG) (100 or 350 IU). Estrus activity was evaluated daily after the application of eCG doses up to day 7. Transrectal ultrasound (US) scanning was performed on days −8 and −2 to confirm anovulation (AN) as well as on days 10 and 45 post eCG treatment, to assess both ovulatory rate (OR) and pregnancy rate (PG), respectively.

The study was carried out in the Comarca Lagunera, Coahuila, northern Mexico (25°51’ North, 103°16’ West, 1190 m), during February and March of the natural anestrus season at this latitude [19,22]. Adult, multiparous (two to three lactations), multiracial, dairy-type goats (Alpine–Saanen–Nubian × Criollo; *n* = 70) managed under intensive, stall-fed conditions were distributed into two homogeneous groups regarding live weight (LW, 41.9 ± 9.08 kg) and body condition (BC, 1.87 ± 0.04; scale from 1 to 4 [26]). The groups were housed in two pens with an area of 80 m^2^ each; fodder was provided three times a day (08:00, 13:00, and 17:00), including alfalfa hay and 200 g per goat/day of commercial concentrate (14% CP). Water and mineral salts were provided ad libitum. During the pre-trial stage, the anestrus status of goats was confirmed through two trans-rectal ultrasound scans, using a 7.5 MHz human prostate transducer (Aloka 500, MHz linear array; Corometrics Medical Systems, Inc., Wallingford, CT, USA). Prior to the ultrasound scan, the transducer was lubricated and then inserted into the goat’s rectum to determine the type of ovarian structures present in both ovaries. Goats with the presence of corpus lutea were discarded from the study. The central activities performed during the experimental period are depicted in Figure 1.

### 2.3. Behavioral Study, Social Rank, Treatment Groups, Measurements, and Response Variables

To determine the goat’s social rank, one month prior to the treatment group formation (i.e., the application of the eCG), a behavioral study was carried out in February as previously outlined [1]. The behavioral test was performed at feeding time (08:00, 13:00, and 17:00) during a 60 min period during the 7 days pre-trial period. Therefore, the main interactions exerted among breeding female goats were monitored for 180 min d^-1^, for a total of 1260 min (i.e., 21 h) during the whole pre-trial behavioral study. The following behavioral goat-to-goat interactions were documented: bumps, threats, shoves, chases, escapes, and evasions. All the agonistic interactions during each 60 min observation period (180 min per day) were recorded; the abovementioned agonistic interactions between two individuals that involved an instigator or a victim, whether or not physical contact occurred, and that resulted in the physical displacement of an animal, were therefore considered. With the information obtained from the agonistic interactions, that is, the result of either winning or being defeated, a success rate (IE) was calculated considering the following formula: IE = number of individuals able to displace/(number of individuals able to displace + number of individuals displaced). According to the obtained IE, goats were classified into three social ranks: low (LSR; IE 0 to 0.33), medium (MSR; IE 0.34 to 0.66) and high (HSR; IE 0.67 to 1) [1,10]. The ethogram considering the observed social hierarchy according to the average agonistic interactions of the response variables during the behavioral test within each social rank are presented in Table 1.

Once the anestrus status confirmation and the social rank was established (i.e., LSR, MSR, HSR), the confirmed social rank groups were returned to the pens; fodder was provided three times a day as described. Then, in mid-March, all goats received one intramuscular dose of 25 mg of progesterone (Progesvit^®^, Brovel, Mexico). One day later, the D100 group (*n* = 35) received 100 IU of eCG per female (Folligon^®^, Intervet, Mexico) while, simultaneously, the D350 group received 350 IU of eCG per female. Both progesterone and eCG doses were applied intramuscularly; the LSR, MSR, and HSR were randomly distributed within each eCG dose group.

Thereafter, the estrus activity was monitored daily from the day of eCG application until day 7 of the experimental period (Figure 1); the evaluation of estrus behavior was carried out twice a day (09:00 and 17:00 h) and lasted 15 min for each evaluation. To identify the estrus activity, a total of seven sexually active males were used; in order to prevent sexual intercourse, each buck was aproned. Previously, bucks were subjected to a hormonal treatment of testosterone to activate their sexual behavior and ensure libido [27]. Once the goat remained immobile and allowed the teaser-buck to mount, the onset of estrus was ruled. Subsequently, the apron was removed from the bucks and the female goats from both experimental groups were exposed to natural mounts for the first 12 h after the onset of the estrus.

The percentage of females in estrus was considered as the number of estrus females/total treated females × 100. Latency to estrus was defined as the time elapsed between the application of eCG and the first mount allowed by the goat. The duration of the estrus was considered as the interval between the first and the last mount allowed by the female. Ovulatory activity was measured 10 d after the application of the eCG, by means of a transrectal ultrasound (Figure 1). The percentage of goats that ovulated was considered as the total of females that ovulated/total treated females × 100. The ovulatory rate was defined as the total number of corpus lutea per group compared to the total number of ovulating goats. The pregnancy rate was evaluated by means of a transrectal ultrasound at 45 d after the application of the eCG. Therefore, the response variables included: estrus induction (EI, %), latency to estrus (LAT, h), duration of estrus (DUR, h), ovulation (OVU, %), ovulation rate (OR, n), corpus luteum size (CLS, cm), pregnancy (PREG, %), kidding (KIDD, %), and litter size (LS, n). Due to the fact that the definition of the social rank status in each goat was individually classified, each goat within the eCG dose treatment group was defined as an experimental unit.

### 2.4. Statistical Analyses

The statistical design was a completely randomized 3 × 2 factorial arrangement with three social ranks (i.e., LSR, MSR, HSR) and two eCG doses (i.e., 100 or 350 IU). The response variables were ANOVA analyzed; the model included the independent variables dose, social rank, and the interaction, with each animal considered as a single experimental unit [28]. In the event of a significant effect, least square mean separation considered the PDIFF option; the analyses were solved by means of the GLM procedures of SAS. Because of their non-normal distribution, categorical variables were analyzed through the CATMOD procedure of SAS. All the analyses were computed through the procedures of SAS (SAS Inst. Inc. Version 9.4, 2016, Cary, NC, USA); the significance level was set at *p* < 0.05.

## 3. Results

### 3.1. Effect of Social Rank and the eCG Dose upon the Response Variables

The dependent variables estrus induction live weight (LW, kg), body condition (BC, units), (EI, %), latency to estrus (LAT, h), duration of estrus (DUR, h), ovulation (OVU, %), ovulation rate (OR, n), corpus luteum size (CLS, cm), pregnancy (PREG, %), kidding (KIDD, %), and litter size (LS, n) as affected by the social rank [R] (i.e., HSR, MSR and LSR) and eCG dose [D] (i.e., 100 or 350 IU), as well as the R × D interaction, are shown in Table 2. 

While LW was affected (*p* < 0.05) by social rank, observing the best LW values in both the HSR and MSR, the lowest LW occurred in the LSR and no LW differences (*p* > 0.05) occurred between eCG doses. Moreover, neither R, nor D or even the R × D interaction affected the phenotypic expression of BC. The response variables OVU and CLS favored (*p* < 0.05) the HSR (96% and 1.04 ± 0.07 cm) and MSR (93% and 1.05 ± 0.07 cm) goats, with the LSR goats showing the lowest values (78% and 0.08 ± 0.09 cm). In turn, an increased (*p* < 0.05) LS occurred in the D350 group (2.06 ± 0.2 vs. 1.36 ± 0.2 cm; no differences (*p* > 0.05; 38.5%) in KIDD occurred among social ranks nor between eCG doses.

### 3.2. Effect of Social Rank × eCG Dose Interaction upon the Response Variables

A rank x dose interaction (*p* < 0.05) affected the response variables LW, EI %, LAT, DUR, OR, and PREG. Therefore, information on such response variables in the HSR, MSR, and LSR as affected by the eCG doses is shown in Table 3. While LW was affected (*p* < 0.05) by the R × D interaction, the best LW values occurred in HSR and MSR and LSR had the lowest LW, the last irrespective of the eCG dose. In addition, the largest (*p* < 0.05) EI % was observed in the HSR goats, irrespective of eCG (i.e., 100 or 350 IU).

**Figure 2:**
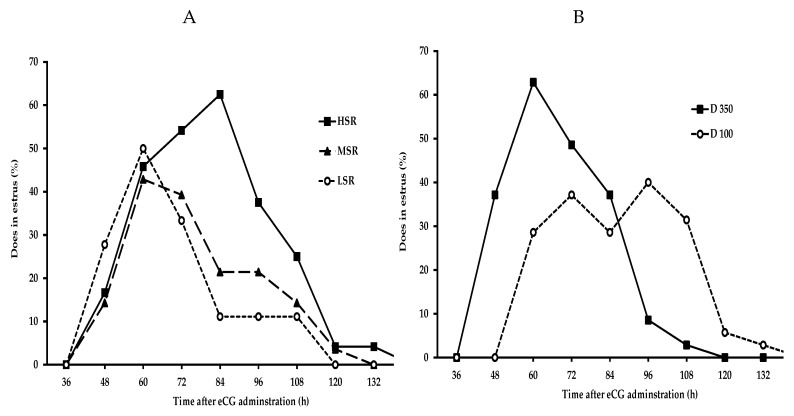
Percentage of estrus induction with respect to the application of equine chorionic gonadotropin (eCG), until the appearance of estrus behavior in goats according to (**A**) social rank (high, medium, and low) and (**B**) eCG dose (100 or 350 IU) in multiracial (Alpine–Saanen–Nubian × Criollo; *n* = 70) dairy goats managed under intensive stall-fed conditions in Northern Mexico (March, 25°51’ North). All goats were primed with progesterone and received different doses of eCG (100 or 350 IU) according to the defined social rank: low, middle, or high.

In general, the greater the eCG dose, the shorter the time it took for estrus to occur; D350 generated the shortest time (*p* < 0.05) for estrus appearance, irrespective of social rank. Regarding the estrus duration (DUR, h), the shortest estrus was shown by the LSR + D100 combination, with no differences among HSR and MSR either with D100 or D350. With respect to ovulation rate, the largest values (*p* < 0.05) were shown by the HSR and MSR within D350, with intermediate values in the HSR, MSR within D100, and LSR + D350; the lowest (*p* < 0.05) OR was shown by the LSR + D100 goats. Regarding the pregnancy rate, the best values (*p* < 0.05) were observed in the HSR + D350, the MSR + D100, and the LSR + D350. Finally, while in the HSR goats the estrus peak occurred 84 h after eCG administration, in the MSR and LSR groups, it occurred at 60 h. With respect to the eCG dose, while the estrus peak in the D350 group occurred 60 h after eCG administration, in the D100 group, it occurred within an interval of 72 to 96 h (Figure 2A,B).

## 4. Discussion

The obtained results support our working hypothesis, in that most of the response variables were positively affected by social rank, favoring to the HSR goats (i.e., EI %, DUR h, OVU %, OR n, and CLS cm). In addition, an increased OR and PREG occurred in the HSR + D350 group, and D350 increased LS, irrespective of social rank. Dominance hierarchies encompass animal societies; social rank is affected by either internal (i.e., body size, body weight, body condition) or external (i.e., parental dominance, previous experiences) factors [29]. Certainly, in many ruminant species, reproductive success and access to food are not shared equally among the members of a herd. While social dominance ensures access to the best available food, it also exerts a positive and significant effect upon live weight. In turn, such an increased live weight enhances the metabolic status while boosting reproductive fitness [30]. Moreover, even when free access to food is available, dominant cues would promote the selection of the most nutritive ration because of a preferential access to food, favoring live weight [31]. Energy balance is a key internal cue for an animal to use in order to decide whether or not to trigger the onset or resumption of reproductive function [32]. In turn, the energy balance will affect both live weight and body condition, as well as influencing reproductive and productive performance either at pre-breeding [33] or pre-partum stages [34], both in males or females [35]. Other studies have found that, besides to live weight, the presence of horns and chronological age also influence social rank [2,4,36].

As commented, increases in both live weight and body condition are known factors inducing reproductive function in anestrus females or enhancing sexual and reproductive outcomes in cycling females [37,38]. So, based in the positive relationship among HSR, increased LW, and augmented BC, these three components will be closely aligned to an increased LH pulse frequency pattern. Such a behavioral neuro-endocrine scenario is prone to encouraging an augmented ovarian function. Our findings agree with previous studies where high hierarchy females had a faster and higher response than low hierarchy females regarding LH pulse frequency and estrus activity [7]. Some biological triggers of such increased ovarian function include reproductive hormones such as follicle stimulating hormone (FSH) or LH [32,39,40], metabolic endocrine cues such as leptin [41], insulin-like growth factor-1 [42], insulin–triiodothyronine [43], the somatotropic GH-IGF-1 axis [44], some defined neuroendocrine hints such as the kiss-1, kisspeptin, gpr-54 complex, kisspeptinergic neurons, and GnRH release [32,45], some other genomic signals such as the OCT2, TTF1, and EAP1 hypothalamic genes [32,46], and even specific nutritional molecules such as glutamate [47,48] and β-carotene [40,49].

Another interesting physio-neuro-endocrine scenario is that the HSR goats, who showed the heaviest LW, may have exerted an increased ovarian function using a non-GnRH-dependent pathway, while involving the potential action not only of diverse intra-ovarian but intra-follicular systems (i.e., the insulin–glucose, IGF-1, and leptin metabolic systems) [50]. Besides, both depressed metabolic status and reduced LW, as that observed in the LSR goats, have been shown to decrease the number of primordial, primary, and Graffian follicle populations, leading to a reduced aromatase mRNA expression and diminished FSH and LH receptors, as well as reductions in leptin levels and Ob-R transcripts [51]. Based on such findings, is tempting to suggest that the HSR and high LW goats may have exerted an enhanced steroidogenesis in those follicles with the lowest threshold to the action of FSH. Such a scenario would increase follicle recruitment, augmenting the aromatization from androgens to estrogens, enlarging the formation and size of the antrum, while increasing oocyte quality, ovulation rate, and fertilization rate. All of them are followed by enhancements in luteogenesis, progesterone synthesis, embryonic implantation, and gestation [32]. Interestingly, an HSR has been related to high androgen levels in males [52], while females treated with estradiol or testosterone displayed less submissive behavior compared to those receiving progesterone or placebo [53].

With respect to the eCG dose (i.e., 100 or 350 IU) the goat response was similar LW (41.1 kg), BC (1.9 units), EI (75.5%), DUR (21.9 h), OVU (90%), CLS (0.98 cm), PREG (53%), and KIDD (38.5%). Our results agree with other studies, where hormonal protocols involving intramuscular progesterone + eCG or hCG were used in anestrus goats [19,22]. Indeed, the eCG + P4 treatment induced reproductive activity in non-cycling goats. The latter was certainly due to the particular function of this gonadotropin, which has a primary activity of FSH and secondarily of LH [18,54]. Therefore, it can be assumed that both doses were sufficient to stimulate the preovulatory LH surge in both eCG groups, promoting not only ovulation but an optimum estradiol level required for estrus induction, ovulation, and pregnancy. However, a reduced LAT with increases in both OR and LS were observed in the D350 group. This was probably due to the fact that a higher number of FSH and LH receptors were expressed in the D359 group, promoting an increased steroidogenesis, an augmented aromatization in theca cells, a larger antrum formation, and a faster peak in estradiol levels, as well as an earlier LH surge, all of which merged to an increased follicular recruitment augmenting both OR and LS in the D350 goats [18].

## 5. Conclusions

This study reveals the key effect of social rank—clearly linked to live weight—upon reproductive outcomes in anestrus goats subjected to an estrus induction protocol primed with progesterone and receiving a differential eCG dose, either 100 or 350 IU, in crossbred dairy goats. Most of the response variables were affected by social rank, favoring to the high social rank goats (i.e., EI %, DUR h, OVU %, OR n, and CLS cm). In addition, a reduced LAT, with increases in OR and LS, occurred in the HSR + D350 group, while D350 increased LS irrespective of social rank. The lowest ranked goats were lighter compared to the MSR and HSR goats, indicating a strong food competition. Interestingly, since no differences regarding EI, DUR, OVU, CLS, PREG, or KIDD occurred between eCG doses (i.e., 100 or 350 IU), the obtained values support the use of a reduced level of exogenous hormones for the induction of estrus during the natural anestrus season. Any attempt to reduce the use of exogenous hormones for the reproductive control of domestic animals will always be welcomed by a highly informed society committed to animal welfare and food safety. These findings are relevant not only from a behavioral, physiological, and wellbeing standpoint, but also acquire productive significance in order to speed up the out-of-season reproductive efficiency of the dairy goat industry.

## Figures and Tables

**Table 1:** Least square means ± standard error for winning events [threats, bumps, shoves, and chases] or lost events [evasions and escapes] in the behavioral study carried out to define the social hierarchy based on a success index according to the average agonistic interactions defining the low (LSR), medium (MSR), or high (HSR) social rank in multiracial (Alpine–Saanen–Nubian × Criollo; *n* = 70) dairy goats managed under intensive stall-fed conditions in Northern Mexico (February, 25°51’ North).

Behaviors	Social Rank		
	LSR	MSR	HSR
**Threats**	25 ± 5.6	106 ± 8.7	231 ± 19.5
**Bumps**	6 ± 1.4	27 ± 3.6	46 ± 7.7
**Shoves**	2 ± 0.5	5 ± 1.0	13 ± 3.2
**Chases**	0 ± 0	0 ± 0	1 ± 0.5
**Evasions**	345 ± 48.1	125 ± 11.1	67 ± 8.9
**Escapes**	1 ± 0.4	0 ± 0	0 ± 0
**Success Index ^1^**	0–0.33	0.34–0.63	0.63–1.0

^1^ Number of individuals able to displace/(number of individuals able to displace + number of individuals displaced).

**Table 2:** Least square means ± standard error for live weight (LW, kg), body condition (BC, units), estrus induction (EI, %), latency to estrus (LAT, h), duration of estrus (DUR, h), ovulation (OVU, %), ovulation rate (OR, n), corpus luteum size (CLS, cm), pregnancy (PREG, %), kidding (KIDD, %), and litter size (LS, n) according to social rank (i.e., LSR, MSR, and HSR) and eCG dose (i.e., 100 or 350 mg) in multiracial (Alpine–Saanen–Nubian × Criollo; *n* = 70) dairy goats managed under intensive stall-fed conditions in Northern Mexico (March, 25°51’ North) ^1.^

Variables	Social Rank (R)			eCG Dose (D)		*p* Value		
	LSR	MSR	HSR	100	350	R	D	R × D
**LW (kg)**	31.6 ± 1.6 ^b^	44.0 ± 1.3 ^a^	49.0 ± 1.4 ^a^	41.9 ± 1.5 ^a^	41.8 ± 1.4 ^a^	0.001	0.615	0.001
**BC (units)**	1.8 ± 0.07 ^a^	1.9 ± 0.06 ^a^	1.9 ± 0.06 ^a^	1.9 ± 0.05 ^a^	1.9 ± 0.05 ^a^	0.768	0.733	0.978
**EI (%)**	10/18 (56) ^b^	20/28 (71) ^b^	23/24 (96) ^a^	26/35 (74) ^a^	27/35 (77) ^a^	0.007	0.599	0.019
**LAT (h)**	57.6 ± 4.6 ^a^	69.6 ± 3.2 ^a^	68.3 ± 3.0 ^a^	76.1 ± 3.0 ^b^	57.7 ± 2.8 ^a^	0.373	0.001	0.001
**DUR (h)**	17.3 ± 4.0 ^b^	18.9 ± 3.2 ^b^	29.0 ± 3.5 ^a^	20.2 ± 2.9 ^a^	23.6 ± 2.9 ^a^	0.004	0.313	0.051
**OVU (%)**	14/18 ^b^ (78)	26/28 ^ab^ (93)	23/24 ^a^ (96)	30/35 ^a^ (86)	33/35 ^a^ (94)	0.05	0.177	0.294
**OR (n)**	1.27 ± 0.17 ^a^	1.77 ± 0.13 ^a^	1.58 ± 0.14 ^a^	1.09 ± 0.10 ^b^	2.06 ± 0.10 ^a^	0.079	0.001	0.001
**CLS (cm)**	0.8 ± 0.09 ^b^	1.05 ± 0.07 ^a^	1.04 ± 0.07 ^a^	0.91 ± 0.06 ^a^	1.05 ± 0.06 ^a^	0.046	0.08	0.13
**PREG (%)**	9/18 ^a^ (50)	14/28 ^a^ (50)	14/24 ^a^ (58)	16/35 ^a^ (46)	21/35 ^a^ (60)	0.651	0.147	0.037
**KIDD (%)**	5/18 ^a^ (28)	10/28 ^a^ (36)	12/24 ^a^ (50)	11/35 ^a^ (31)	16/35 ^a^ (46)	0.279	0.184	0.119
**LS (n)**	1.60 ± 0.4 ^a^	1.80 ± 0.3 ^a^	1.83± 0.2 ^a^	1.36 ± 0.2 ^b^	2.06 ± 0.2 ^a^	0.228	0.023	0.139

^1^ In February, a behavioral study was carried out to define the social ranks; low (LSR), middle (MSR), or high (HSR) social rank. Then, all goats were exposed to an estrus induction protocol to induce reproductive activity (March). All goats were primed with progesterone (P4) and received different doses of eCG (100 or 350 IU). ^a,b^ Least square means without a common superscript within a response variable are different (*p* < 0.05).

**Table 3:** Least square means ± s.e. for live weight (LW, kg), estrus induction (EI, %), latency to estrus (LAT, h), duration of estrus (DUR, h), ovulation (OVU, %), ovulation rate (OR, n), corpus luteum size (CLS, cm), pregnancy (PREG, %), kidding (KIDD, %), and litter size (LS, n) as affected by the social rank (i.e., HSR, MSR, and LSR) × eCG dose (i.e., 100 or 350 mg) interaction in multiracial (Alpine–Saanen–Nubian × Criollo; *n* = 70) dairy goats managed under intensive stall-fed conditions in Northern Mexico (March, 25°51’ North) ^1.^

Variables	eCG-100				eCG-350	
	LSR	MSR	HSR	LSR	MSR	HSR
**LW (kg)**	30.0 ± 2.4 ^b^	43.4 ± 1.8 ^a^	47.6 ± 1.9 ^a^	33.0 ± 2.1 ^b^	44.7 ± 1.8 ^a^	47.4 ± 2.1 ^a^
**EI (%**)	3/8 ^c^ (38)	11/14 ^ab^ (79)	12/13 ^ab^ (92)	7/10 ^ab^ (70)	9/14 ^bc^ (64)	11/11 ^a^ (100)
**LAT (h)**	72.0 ± 8.4 ^abc^	79.6 ± 4.4 ^a^	74.0 ± 4.2 ^ab^	51.4 ± 5.5 ^d^	57.3 ± 4.8 ^cd^	62.2 ± 4.4 ^bcd^
**DUR (h)**	7.5 ± 5.9 ^b^	18.9 ± 4.5 ^ab^	29.5 ± 4.6 ^a^	25.2 ± 5.3 ^a^	18.9 ± 4.5 ^ab^	28.4 ± 5.0 ^a^
**OR (n)**	0.86 ± 0.3 ^d^	1.2 ± 0.2 ^cd^	1.2 ± 0.2 ^cd^	1.6 ± 0.2 ^bc^	2.3 ± 0.2 ^a^	2.1 ± 0.2 ^ab^
**PREG (%)**	3/8 ^b^ (38)	9/14 ^ab^ (64)	4/13 ^b^ (31)	6/10 ^ab^ (60)	5/14 ^b^ (36)	10/11 ^a^ (91)

^1^ In February, a behavioral study was carried out to define the social ranks; low (LSR), middle (MSR), or high (HSR) social rank. Then, all goats were exposed to an estrus induction protocol to induce reproductive activity (March). All goats were primed with progesterone (P4) and received different doses of eCG (100 or 350 IU). ^a–d^ Least square means without a common superscript within a response variable are different (*p* < 0.05).
